# Sediment Facilitates Microbial Degradation of the Herbicides Endothall Monoamine Salt and Endothall Dipotassium Salt in an Aquatic Environment

**DOI:** 10.3390/ijerph15102255

**Published:** 2018-10-15

**Authors:** Md. Shahidul Islam, Trevor D. Hunt, Zhiqian Liu, Kym L. Butler, Tony M. Dugdale

**Affiliations:** 1 Centre for AgriBioscience, Agriculture Victoria, 5 Ring Rd, La Trobe University, Bundoora 3088, Australia; Shahidul.Islam@ecodev.vic.gov.au (M.S.I.); trevor.hunt@ecodev.vic.gov.au (T.D.H.); zhiqian.liu@ecodev.vic.gov.au (Z.L.); 2 Biometrics Unit, Hamilton Centre, Agriculture Victoria, 915 Mt Napier Rd, Hamilton 3300, Australia; kym.butler@ecodev.vic.gov.au

**Keywords:** mesocosm, irrigation canal, irrigation channel, biodegradation, persistence, aquatic weed

## Abstract

Endothall dipotassium salt and monoamine salt are herbicide formulations used for controlling submerged aquatic macrophytes and algae in aquatic ecosystems. Microbial activity is the primary degradation pathway for endothall. To better understand what influences endothall degradation, we conducted a mesocosm experiment to (1) evaluate the effects of different water and sediment sources on degradation, and (2) determine if degradation was faster in the presence of a microbial community previously exposed to endothall. Endothall residues were determined with LC-MS at intervals to 21 days after endothall application. Two endothall isomers were detected. Isomer-1 was abundant in both endothall formulations, while isomer-2 was only abundant in the monoamine endothall formulation and was more persistent. Degradation did not occur in the absence of sediment. In the presence of sediment, degradation of isomer-1 began after a lag phase of 5–11 days and was almost complete by 14 days. Onset of degradation occurred 2–4 days sooner when the microbial population was previously exposed to endothall. We provide direct evidence that the presence and characteristics of sediment are of key importance in the degradation of endothall in an aquatic environment, and that monoamine endothall has two separate isomers that have different degradation characteristics.

## 1. Introduction

Endothall is a herbicide used for controlling submerged aquatic weeds and algae. Endothall is available in two formulations: Endothall dipotassium salt and endothall dimethylalkylamine salt (hereafter monoamine endothall). The active ingredient for both formulations is endothall acid (7-oxabicyclo[2.2.1]heptane-2,3-dicarboxylic acid), which is free when endothall salts are applied to water [1]. Endothall is a contact herbicide that severely affects plant physiological processes, disrupting plant cell membranes [2,3,4,5], damaging plant tissues within 2–5 days after application [6,7]. Endothall is highly effective against many submerged aquatic weed species [1,8,9,10,11,12,13,14]. Endothall has been widely used in the USA for decades [15,16], has been registered in New Zealand [17], and registration is currently being pursued in Canada and Australia. Both formulations of endothall are used to control nuisance aquatic vegetation in a variety of aquatic systems including ponds, lakes, pools and irrigation channels [18,19,20]. 

In terrestrial situations, herbicides are typically applied directly to the foliage of weeds, where absorption occurs and subsequently the weed dies. In contrast, when endothall is used to control submersed aquatic weeds, it is applied to the water column in which the weeds are growing to achieve a target concentration throughout the water column in the target area. Absorption of endothall into the foliage then occurs from the water. A consequence of this application method is that all other non-target organisms in the water are exposed to endothall. 

An emerging use-pattern is the application to flowing irrigation canals to remove submersed weeds that obstruct flow. In this use, the target concentration is achieved by metering a volume of endothall into the canal at a rate proportional to the discharge for a designated period to provide the target exposure time. This pulse of endothall-treated water then moves down the irrigation canal. As irrigation canals have many connections to natural rivers and wetlands, this endothall-treated water can discharge into other aquatic ecosystems, or it can be irrigated directly onto crops.

The concentration of endothall in the water and the length of time plants are exposed to endothall (exposure time) determine the effectiveness of endothall against the target weeds [15,21] and the toxic effect on any non-target organism. Once applied, the aquatic ecosystem is exposed to endothall until it is completely removed by natural degradation processes or dissipation. Therefore, the length of time required for complete degradation of endothall is critical for the safety of non-target organisms and any other uses of the water or aquatic ecosystem (e.g., agriculture, recreation, drinking). Endothall degradation rate and therefore, persistence time, can vary greatly among different systems, depending on the prevailing physical, chemical and hydrodynamic properties [16,22,23,24]. Persistence time has been reported to differ greatly between the formulations of endothall [7], therefore, it is essential to improve our understanding of the patterns and processes of degradation of both formulations of endothall to better predict the persistence time in aquatic ecosystems.

Most studies on endothall dissipation and persistence were conducted during the 1960s and 1970s, following the first aquatic use registration of endothall in 1960 in the USA, and the results have been reviewed and summarized elsewhere [16,22,23,24]. Most of those studies focused on a single formulation of endothall, mainly the dipotassium salt. A major limitation in most previous studies is that the endothall concentrations in water were analyzed using a bioassay technique originally developed in 1962 [25], which is indirect and less precise than modern quantitative techniques such as LC-MS [26]. In addition, none of these early studies systematically considered the effects of environmental variables on endothall persistence. 

The transformation and degradation of endothall in aquatic environments is performed by microbes, particularly bacteria [26,27], and the loss of endothall by other means such as volatilization, sorption, photolysis, hydrolysis and oxidation are negligible [16,23,28]. Earlier studies suggest that provision of an environment that supports microbial growth will enhance the rate of endothall degradation [25,26,27]. Arthrobacter bacteria isolated from a lake hydrosoil have been shown to use endothall as the sole source of carbon and energy [26]. 

In this paper, we present the results of an experiment conducted in mesocosms under controlled conditions to determine the effects of different sources of water and sediment on the degradation of monoamine endothall and dipotassium endothall. The experiment was designed to (1) evaluate the effects of three different water and sediment sources on endothall degradation, and (2) determine if degradation was faster in the presence of a microbial community that had previously been exposed to endothall. 

Together, these experiments represent an important advancement because it is the first study that uses a fully replicated experimental design coupled with direct quantification (LC-MS) to systematically determine how (1) presence or absence of sediment, (2) a range of native sediment and non-sterile water sources, (3) formulation of endothall, and (4) pre-exposure of microbial communities to endothall, jointly impact the degradation of endothall. This randomized design allows us to undertake a cause and effect analysis of the effects of these environmental variables on the decay of both formulations of endothall. The methodology provides waterbody managers with reliable information on the effect of environmental variables on decay of contemporary, commercially available, formulations of endothall, and thus enables them to manage endothall-treated water in an informed manner.

## 2. Materials and Methods

### 2.1. Experimental Design 

An experiment was established to examine the effects of water and sediment variables on endothall persistence. The experiment was conducted in mesocosm systems created by filling plastic mesocosms with different combinations of water, sediment and aquatic plants, collected from different sources. The factors and levels are shown in Table 1. The sources of water, sediment and plants were: (i) Melbourne city potable water with and without garden soil (Greensborough, Melbourne, Victoria); (ii) Central Goulburn Irrigation District (Victoria) irrigation channel water with and without sediment; and (iii) Coleambally (New South Wales) irrigation channel water with and without sediment. Ribbon weed plants (*Vallisneria australis* S.W.L. Jacobs and Les) were collected from each irrigation district and included in the treatments with sediment from irrigation channels. 

A total of 72 mesocosms (24 treatments × 3 replicates) with 18 L capacity [(240 × 240 × 300 mm), black, polypropylene, square bucket, Garden City Plastics FV300] were numbered serially from 1 to 72 and randomly assigned to each of the treatments and replicates, i.e., a fully randomized design. Designated mesocosms were first supplied with 2 L of sediment from the respective sediment source and then 12 L of water was added. Mesocosms not containing sediment were filled with 14 L of water only. Three treatments each had two 50 mL centrifuge tubes half filled with the same sediment and pushed into the sediment substrate at the base of the mesocosms. The tubes with sediments remained in situ until collected as sediment samples later during the experiment. Mesocosms containing irrigation channel water and sediment were then supplied with a single ribbon weed plant suspended in the water column. 

To generate a microbial population pre-exposed to endothall, a mesocosm was created three months prior to the experiment. To create this mesocosm, sediment was added to a 100 L polyethylene tank to a depth of ~100 mm, covered with ~50 L of water and two ribbon weed plants added (all sourced from the Central Goulburn Irrigation District). The sediment and water were not autoclaved. Monoamine endothall was applied to a target concentration of 2.4 mg ae L^−1^ and then the tank was left in a glasshouse for three months. The endothall concentration and degradation in the mesocosm was not measured, nor was any additional carbon source added. Treatments that included augmentation with microbes pre-exposed to endothall were dosed with 100 mL of water and 50 mL of sediment from this tank (as a mixed slurry), after addition of water and sediment to the experimental mesocosms. Hereafter, these treatments are referred to as “augmented” versus all the other treatments that are “non-augmented”. 

The mesocosms were placed in a row along the length of a temperature controlled (~18 °C) glasshouse at floor level. Each bucket was individually aerated, such that it created a gentle movement within the water column only, and without agitating the sediment surface. Each air line was fitted with a reverse flow valve to prevent water flowing back into the air delivery system. The mesocosms were left to stabilize for one week before endothall was added. Herbicide, dipotassium endothall (Cascade™) or monoamine endothall (Teton™), was applied to achieve a target concentration of 2.4 mg ae L^−1^, by pouring the required volume of stock solution into each of the designated mesocosms.

### 2.2. Endothall Sampling

#### 2.2.1. Water Column

To determine the concentration of endothall in each mesocosm, 25 mL water samples were collected 0, 1, 3, 5, 7, 9, 11, 16, and 21 days after endothall application. Day-0 samples were collected 3 to 4 h after endothall application, to provide an indication of starting concentration. The sampling times were chosen to end at 21 days because the current permit for endothall use in Australia (issued by the Australian Pesticide and Veterinary Medicines Authority) specifies a 21-day withholding period before the endothall-treated water can be used for watering livestock, preparing agricultural sprays and irrigation. Therefore, it is important to know what the likely endothall concentration is at this time. The transitional sampling times were compressed in the first 12 days because prior studies indicate that most endothall usually degrades by 7 to 14 days [29,30]. 

Water samples were collected from the middle of the water column using a 50 mL syringe fitted with a plastic tube at the tip of the syringe. Each mesocosm had a dedicated sampling syringe and tube which remained in the mesocosm for the duration of the experiment. Samples were acidulated with one drop of 40% HCl solution and stored in a refrigerator until analysis. 

For the treatments with endothall added, endothall acid concentration was determined by LC-MS. Chromatographic separation of endothall was achieved using an Eclipse XDB-C8 column (150 × 2.1 mm, 3.5 µm, Agilent Technologies, Santa Clara, CA, USA) on a Vanquish UPLC system (Thermo Scientific™, Waltham, MA, USA). The mobile phase was composed of 0.5% formic acid (A) and acetonitrile containing 0.1% of formic acid (B). The flow rate was 0.25 mL/min with a gradient elution of 5 to 60% B over 10 min. The injection volume was 5 µL. 

The detection of endothall was by LTQ-Orbitrap Elite mass spectrometer (Thermo Scientific™) operated in electrospray ionization (ESI) negative Fourier transform mode. The heated capillary was maintained at 350 °C with a source heater temperature of 300 °C, and the sheath, auxiliary and sweep gases were at 40, 15 and 5 units, respectively. The source voltage was set to 3.2 kV and the resolution was set to 60,000. Endothall (deprotonated ion) was extracted from a full scan spectrum and quantified using an external calibration curve.

#### 2.2.2. Sediment 

One of the two tubes containing sediment, that were placed at the bottom of selected treatments (Table 1) was removed 7 days after endothall application, and the other tube was removed 16 days after application. Upon removal, excess water was tipped out of the tubes, the tube was then capped and stored at −18 °C. The residual water in the sediment was collected after centrifugation and the level of endothall in the water determined by LC-MS. To verify the possibility of endothall being adsorbed to the sediment, the sediment was washed with 60% acetonitrile solution and the concentration of endothall in the washing liquid was measured by LC-MS. 

At the end of the experiment, sediment samples from all sources and from the control treatments (not exposed to endothall) were collected in 250 mL plastic containers and stored in a refrigerator until they were analyzed at a commercial laboratory.

### 2.3. Water Quality Monitoring

Electrical conductivity (EC, µS/cm^2^), dissolved oxygen (DO, mg ae L^−1^), turbidity (NTU) and pH were measured three times per week by collecting a small volume (25–40 mL) of water from each treatment. EC, DO and pH, were measured using a water quality meter (Hach HQ40D Portable Multi Meter; HACH COMPANY, 389 Loveland, CO, USA), and turbidity was measured using a turbidity meter (Hach 2100Q Portable Turbidimeters; HACH COMPANY, 389 Loveland, CO, USA). A temperature logger was installed in one mesocosm. 

### 2.4. Data Analyses

Two isomers of endothall were detected in the samples (see Results for description), which had different degradation dynamics. Therefore, analyses were split into isomer-1 and isomer-2.

#### 2.4.1. Isomer One 

Statistical analyses were carried out using the 15 treatments with endothall applied (Table 1).

A general pattern for the responses over time (days after endothall application) of each mesocosm was for the response to be reasonably stable at the start of the experimental period (i.e., only a small difference between the concentration at day 0 and day 1) and near the end of the experimental period (i.e., only a small difference between the concentration at day 16 and day 21), but sometimes large and quick (e.g., ~90% reductions in ~2 days) changes between these times. During times of large and quick changes, for mesocosms in a treatment, the between mesocosm variability of the isomer-1 concentration increased substantially, which makes statistical analysis less straightforward. Near the end of the experiment, one treatment, namely treatment 14, was not stable (i.e., the isomer-1 concentrations were declining quickly from days 16 to 21), and hence the isomer-1 concentration at day 21 had much greater variability between mesocosms than the between mesocosm variability for other treatments. Therefore, the treatment 14 mesocosms were not included in some statistical analyses, and in these cases the individual concentrations for the three mesocosms of treatment 14 were presented instead. 

The isomer-1 response curves were statistically examined by calculating and analyzing the following five summary statistics, that have been carefully chosen to summarize the majority of treatment differences in the isomer-1 response curves with monoamine or dipotassium endothall:

1. Isomer-1 concentration at day-0
2. Percent decline in the isomer-1 concentration over the 21-day period (i.e., 100 × (isomer-1 concentration at day-0 − isomer-1 concentration at day-21)/(isomer-1 concentration at day-0)
3. Days to 25% of observed reduction (i.e., number of days since application on the first day when the isomer-1 concentration was first observed to achieve at least a 25% reduction of the full isomer-1 concentration reduction between day-0 and day-21)
4. Days to 50% of observed reduction (i.e., number of days since application on the first day when the isomer-1 concentration was first observed to achieve at least a 50% reduction of the full isomer-1 concentration reduction between day-0 and day-21)
5. Days to 75% of observed reduction (i.e., number of days since application on the first day when the isomer-1 concentration was first observed to achieve at least a 75% reduction of the full isomer-1 concentration reduction between day-0 and day-21)

The days to 25, 50, or 75% of observed reduction can only take on the values 1, 3, 5, 7, 9, 11, 16 or 21 days. 

The isomer-1 concentration at day-0 and the percent decline measure key components of the isomer-1 concentration at day-21. The days to 25%, 50% and 75% reduction collectively measure the relative speed of breakdown of isomer-1.

Each measurement was analyzed using an analysis of variance with mesocosm as the unit of analysis, and that included factorial and nested treatment terms that elucidated the effects present. The isomer-1 concentration at day-21 was analyzed after the mesocosm data had been log(y + 0.3) transformed, and the percent decline in the isomer-1 concentration over the 21-day period was log(1.1 − (y/100)) transformed. These transformations prevented the residual variation being substantially different between treatments with large differences in isomer-1 concentrations near the end of the study period. Additionally, the treatment 14 mesocosms were excluded from these two analyses, for the reason explained earlier. There was no reason to transform the data for the concentration at day-0, or to exclude the mesocosms from treatment 14, and thus neither of these were carried out for this measurement.

The analyses of days to 25%, 50% and 75% reduction were restricted to mesocosms from endothall treatments that had sediment added because these were the only endothall treatments with large reductions in isomer-1 concentrations by day-21. Since these measurements only allowed a restricted number of concentrations (i.e., data was discrete), non-parametric analyses of variance were carried out. Since the times between measurement intervals were not equally spaced, which tends to strongly negate equal variation between treatments, the data was firstly ranked with ties. These ranked data were analyzed using standard analysis of variance methodology. However, *p* values were obtained from the usual F values using a permutation test with 100,000 random permutations.

When calculating F values, the residual mean square was calculated from the saturated model (i.e., a model including an effect for all treatments included in the analysis). Additionally, the standard errors of difference were calculated using the residual mean square from the saturated model.

Statistical analyses were calculated using GenStat 18 [31], including the ANOVA (generally balanced analysis of variance) and FIT (general linear models, not necessarily balanced) directives and the RANK (ranking data) and APERMTEST (permutation tests in analysis of variance) procedures.

#### 2.4.2. Isomer Two

Isomer-2 concentrations were very low in the mesocosms that received dipotassium endothall over the entire study period, and consequently the statistical analyses are restricted to the nine treatments that received monoamine endothall (i.e., treatments 7 to 12 and 22 to 24). Similar to isomer-1, the isomer-2 response curves were statistically examined by calculating and analyzing the following two summary statistics, that have been carefully chosen to summarize the majority of treatment differences in the isomer-2 response curves with monoamine endothall:

1. Isomer-2 concentration at day-0
2. Percent decline in the isomer-2 concentration over the 21-day period (i.e., 100 × (isomer-1 concentration at day-0 − isomer-1 concentration at day-21)/(isomer-1 concentration at day-0))

Similar to isomer-1, each measurement was analyzed using an analysis of variance with mesocosm as the unit of analysis, and that included factorial and nested treatment terms that elucidated the effects present. At day-21 the mesocosms from the three treatments with sediment added, but no augmentation, appeared not to be stable. Hence the isomer-2 concentration at day-21 had much greater variability between mesocosms in these three treatments, than the other six treatments. This greater variability was also reflected in the percent decline in the isomer-2 concentration over the 21-day period. To address this issue, the isomer-2 concentration at day-21 and the percent decline in the isomer-2 concentration over the 21-day period were analyzed using non-parametric analyses of variance, using a similar technique to the ‘speed of decline’ summary measurements for isomer-1. Similar to the isomer-1 concentration at day-0, the isomer-2 concentration at day-0 was analyzed using a parametric analysis of variance, without any transformation.

Since, by day-21, the isomer-2 concentrations had stabilized to a low concentration for only three treatments, the analyses of days to 25%, 50% and 75% reduction provided limited information on the ‘speed of decline’ for isomer-2, thus, analyses are not reported.

As with isomer-1, all statistical inference used the residual mean square from the saturated model. Statistical analyses were calculated using GenStat 18 [31], including the ANOVA directive and the RANK and APERMTEST procedures.

## 3. Results

**Figure 1:**
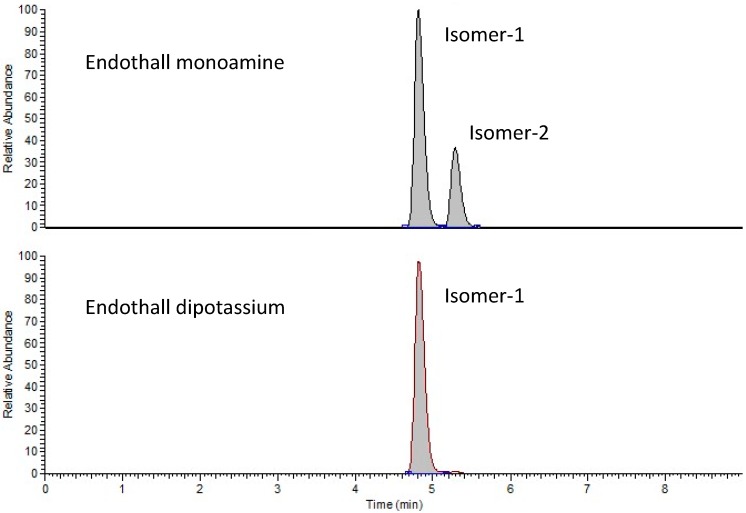
Extracted ion chromatogram of endothall, in two formulations. Endothall concentrations of isomer-1 are 10 mg L^−1^ in both cases.

It is known that endothall exists as a mixture of three stereoisomers of which the (1*R*,2*S*,3*R*,4*S*)-isomer is the most herbicidally active [32]. The number and identity of isomers in endothall herbicide formulations have never been reported to our knowledge. Two isomers of endothall were detected in this study. Isomer-1 was dominant in both endothall formulations, while isomer-2 was present in substantial amounts in monoamine endothall but at trace levels in dipotassium endothall (Figure 1). The differences in isomer composition between these two formulations have not been described before.

### 3.1. Water Quality and Sediment Properties

Water temperature, pH and dissolved oxygen in the mesocosms were similar between the treatments (Table 2) and represent an environment suitable for aerobic microbial growth. Turbidity and electrical conductivity varied by water source and addition of sediment (Table 2).

The sediments from the irrigation channels were both clays, with low organic matter and pH, in contrast to the garden soil, which was a loam with high organic matter and neutral pH (Table 3). Another important difference was that the garden soil was much more biologically active (as measured by total colony forming units per g soil; Table 3).

### 3.2. Endothall Degradation

**Figure 2:**
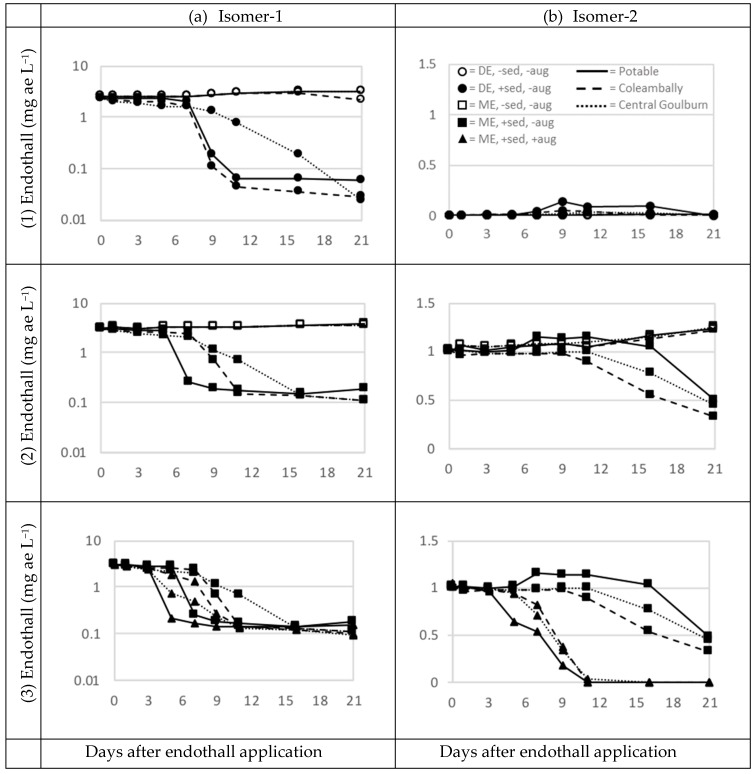
Endothall isomer-1 (**a**) and isomer-2 (**b**) concentrations in mesocosms for each treatment. Values represent average over three replicates. Plots represent various combinations of endothall formulation, source of water and sediment, presence or absence of sediment, and microbe augmentation. Note different *y*-axis scales between left and right panels. (1) Treatments containing dipotassium endothall without microbe augmentation. (2) Treatments containing monoamine endothall (without microbe augmentation). (3) Treatments containing monoamine endothall with sediment, ±microbe augmentation. Note: a subset of treatments is shown in both (2) and (3). Legend provided in top right panel: Water sources represented by line style; endothall form (DE = Dipotassium endothall, ME = Monoamine endothall), sediment presence or absence (±sed) and pre-exposure to endothall (±aug), represented by shape and full of symbols.

Endothall (isomer-1) persisted for the 21-day duration of the experiment in the treatments that did not contain sediment. In contrast, where sediment was present, there was an initial period of endothall persistence, followed by rapid degradation commencing by days five to seven. After this period of rapid degradation, endothall concentration remained above zero (Figure 2). Isomer-2 followed the same trend in relation to sediment presence for treatments that contained monoamine endothall, although it did not always degrade to low levels, even in the presence of sediment.

At day-0, the starting concentrations of monoamine endothall (isomer-1) only differed with endothall formulation and presence of sediment (Table 4). The starting concentrations were 3.05 (sediment present) and 3.08 (sediment absent) mg ae L^−1^, and of dipotassium endothall were and 2.27 (sediment present) and 2.50 (sediment absent) mg ae L^−1^, with the standard error of difference ranging between 0.038 and 0.044.

During the 21-day period of the experiment endothall did not degrade in water without sediment; its concentration increased by 19% and 24% for monoamine and dipotassium endothall, respectively (Table 5 and Table 6). These increases in concentration were likely due to evaporation of water from the mesocosms. In contrast, 96% of monoamine endothall (with or without augmentation) had decayed by day-21, and 98% of dipotassium endothall had decayed by day-21 in the treatments with sediment added (Table 5 and Table 6; Figure 2). The endothall remaining was lower when sediment was sourced from irrigation channels (97% reduction) than from a garden (96% reduction, Table 6). Endothall decline was also slightly greater with sediment that was augmented with sediment slurry pre-exposed to endothall than when it was not augmented (Table 6; Figure 2).

Degradation of isomer-2 in monoamine endothall treatments was similar to isomer-1, i.e., it decayed only when sediment was added and to a greater degree when sediment was augmented with sediment slurry pre-exposed to endothall (Figure 2). Notable differences were that (1) there was no effect of sediment on the concentration at day-0 (F = 1.71, *p* = 0.21), (2) the concentration of endothall remaining at day-21 was not affected by source of water or sediment (Table 7), and (3) that isomer-2 decayed to zero when the sediment was augmented (Table 8), which did not occur for isomer-1 in any treatments (Table 6).

Mean endothall isomer-1 and isomer-2 concentrations in the sediment were 0.07 (range 0.01–0.17), and 0.23 (range 0.04–0.45) mg ae L^−1^, respectively, indicating that endothall did not accumulate in the sediment.

### 3.3. Rate of Endothall Degradation

For the treatments that included sediment, degradation of endothall was characterized by a period of slow degradation followed by rapid degradation to near zero endothall (Figure 2). In this situation, the time to first observing 25%, 50% and 75% of the observed degradation can be used to characterize both the onset of degradation and the rapidity of degradation during the rapid degradation period.

Degradation was earlier when sediment was augmented with microbes, by up to 4 days (Table 9 and Table 10). Monoamine endothall with microbe augmentation decayed by 25% by day-5 for all water sources, while the treatments without augmentation (amine and dipotassium endothall) took 5–9 days to reach this threshold. Likewise, most treatments with pre-exposure to endothall reached 50 and 75% degradation 2–4 days before unaugmented treatments (Table 9). Whilst potable water + garden sediment did not necessarily lead to earlier degradation, once the rapid degradation phase had commenced the degradation in potable water + garden sediment was substantially more rapid than in irrigation water. In fact, the predicted day for first observing 25% reduction was the same as the predicted day for first observing 75% reduction in all garden soil treatments (Table 9). In general, dipotassium endothall reached these degradation thresholds at the same time as monoamine endothall, except in potable water + garden sediment, where it decayed to these thresholds two days later (Table 9). Onset of degradation for isomer-2 occurred several days later than isomer-1 and decayed to trace concentrations only when augmented with sediment slurry pre-exposed to endothall (Figure 2).

## 4. Discussion

The presence of the two isomers of endothall have not been reported previously. We have found these in similar relative proportions in all batches of dipotassium and monoamine endothall that we have characterized with LC-MS, from laboratory and field studies of endothall. The concentration of isomer-1 correlates with the concentration of endothall measured by ELISA (RaPID Assay^®^ Endothall Test Kit, Strategic Diagnostics Inc.; authors unpublished data) and correlates closely with the target concentration in the water column after dosing. The relative herbicidal potency of these two isomers remains unknown and it is puzzling that isomer-2 is present in a substantial proportion only in the monoamine formulation. To properly understand the degradation of endothall it is critical that we know what its component parts are and their degradation dynamics. Our data show that the two isomers showed clear differences in environmental fate, i.e., isomer-2 was much more persistent than isomer-1 (Figure 2). However, our experiment was not designed to determine the degradation products of endothall and we saw no evidence that isomer-1 was converted to isomer-2. In common with isomer-1, the degradation of isomer-2 was influenced by presence of sediment and augmentation with microbes.

The environmental conditions in the mesocosms were aerobic and suitable for microbial growth, demonstrated by the biological activity that was recoded from the sediment substrate (Table 2 and Table 3). Therefore, degradation of endothall should occur in all mesocosms, but persistence of endothall varied greatly according to the environmental factors that were manipulated in the mesocosms. The presence or absence of a sediment substrate had the largest influence on endothall persistence. When no sediment substrate was present there was no degradation detected over the 21 days, and this was consistent with all three water sources tested. We did not detect any accumulation of endothall in the sediment, therefore we surmise that the removal of endothall from the water column was due to a process of degradation, rather than adsorption to soil. Hiltibran [25] found that endothall (disodium salt) applied at 5 mg ae L^−1^ took 40–61 days to reach 0.1 mg L^−1^ in lake water in the absence of sediment, in contrast to 13 days when mud was added to the same lake water. The same study also compared tap water which alone took 21 days to reduce endothall from 1.0 to 0.1 mg L^−1^ but the same water took eight days with the addition of mud. Our results, which are based on a fully replicated experimental design that used LC-MS to determine endothall residues, corroborate the findings of Hiltibran [25], who determined disodium salt endothall residues from unreplicated mesocosms using a flaxseed bioassay method, and extend them to the two formulations of endothall that are currently used to manage aquatic weeds.

Since we concluded our experiment after 21 days, we are not able to suggest what would happen to the endothall in water-only treatments beyond this 21-day time frame. It is, however, obvious that the non-sterile water itself has very little or no effect on endothall degradation, and that endothall persists longer in water without the presence of a sediment substrate. Degradation will occur whenever sediment or soil is present, even when the soil is taken from a residential garden, suggesting either or both of the following: (1) The microbe(s) responsible for degradation are commonly present in soil and are cosmopolitan; (2) there are a wide range of microbial taxa which can degrade endothall. Despite a number of studies that have investigated endothall degradation in the presence of a range of microbial inoculum from a range of sources [25,27,30], none have attempted to determine if the taxa responsible for degradation are the same when endothall is degraded in different habitats (or in mesocosms with constituents collected from different habitats). The only two studies that report on attempts to isolate the microbes responsible for degradation both found *Arthrobacter* are able to utilize endothall as their sole source of carbon [26,33]. The validity of postulates 1 and 2, above, can be verified by undertaking experiments to identify and isolate the taxa which are responsible for degradation (e.g., time series analysis of microbial community response, enrichment culture techniques and isotopic labelling).

The water collected from irrigation channels and used in these mesocosms was turbid (mean turbidity 22.0 NTU in both water sources) and contained suspended particles, so it was likely to contain a microbial population. Despite this, endothall degradation did not occur in the water column, in our study or that of Hiltibran [25]. We do not know what aspect of the sediment allows degradation to occur, possible explanations include provision of microbial inoculum, a nutrient source, or a habitat substrate for microbes. Assuming degradation occurs on or in the sediment, this finding suggests the possibility that degradation will be proportional to the depth of water overlaying the sediment (i.e., degradation will be greater where the water volume to sediment surface area ratio is low, such as occurs in shallow water bodies).

Where degradation occurred, it began after a lag phase of 5–9 days, depending on treatment combination. Degradation was slow during the lag phase, followed by a relatively rapid decline in endothall concentration until a stable concentration was achieved. However, this low stable concentration was not always achieved in the 21-day duration of the experiment. This lag phase probably reflects a lag phase in the growth of the microbes responsible for degradation, which is typical of microbial growth. Holmberg and Lee [29] reported a similar pattern of degradation of dipotassium endothall applied in a pond (average depth 1 m) at a rate of 5 mg L^−1^. Endothall disappeared slowly for the first 12–13 days, followed by a sharp decline to less than 1 mg L^−1^ during the next few days, and reaching the level of detection in 18 days. A slightly different degradation pattern was reported by Sikka and Rice [30], who found that endothall (dipotassium salt) concentrations decreased in the water column following treatment at 2 mg L^−1^, with a corresponding increase in the top inch of the hydrosoil up to 22 days after treatment. Accumulation in the hydrosoil was rapid during the first 3 days, becoming more gradual through day 22; it was suggested that the initial rapid decline in the water column was because of sorption to the hydrosoil. Endothall disappeared completely from the hydrosoil in 44 days after treatment. In our study, dipotassium endothall concentration was lower by a small amount (0.25 mg ae L^−1^) a few hours after application for the mesocosms which contained sediment. It is possible that this was due to adsorption, like that reported by Sikka and Rice [30], but this cannot be confirmed as we did not determine endothall residues in the sediment until day-7 and day-16 (at which times mean pore water concentrations were 0.09 and 0.05 mg L^−1^, respectively, which were 6 and 33% of the concentration in the mesocosms).

Degradation of isomer-1 occurred at a similar rate between the two formulations, at least for irrigation water, although the dipotassium endothall persisted at a concentration lower than monoamine endothall (0.02–0.06 versus 0.1–0.2 mg ae L^−1^, respectively, Figure 2). For monoamine endothall, this level is only marginally below the minimum dose specified on the product label (0.3 mg ae L^−1^; [34]). Nevertheless, as found in this study, the general characteristics of degradation of dipotassium and monoamine endothall are similar. Only trace concentrations of isomer-2 were present in dipotassium endothall so we did not compare degradation of isomer-2 between the formulations. We do not know of any study that compares the degradation of the two formulations.

It is concerning that endothall did not degrade fully during this study because, if this also occurs in the field, it indicates there is potential for chronic, low concentration exposure to plants after field applications. This persistence is unlikely to be detected in field applications because dissipation and dilution will occur in natural water bodies. Nevertheless, further mesocosm studies of longer duration are required to determine how long it takes for endothall to degrade completely.

Augmentation with a sediment and water slurry from a tank previously exposed to endothall resulted in a significantly shorter lag phase and quicker onset of rapid degradation of isomer-1 of monoamine endothall, typically 2–4 days sooner than treatments without augmentation. With isomer-2, it was observed that the 21-day decline increased from about 50 to 100% with augmentation. It is likely that these effects occurred because the microbial taxa had evolved to utilize endothall during their single previous exposure to it. A similar finding has been reported, where bacteria capable of degrading endothall were isolated from soil as late as one year after treatment, but not from untreated soil [30]. These data indicate that a single exposure to endothall is enough for the microbial community to adapt to using endothall as a carbon source and that repeated use of endothall at a single location may result in faster decay. An undesirable aspect of this is that it could reduce endothall efficacy where long exposure times are important. This is supported by reports that endothall efficacy has reduced after successive application of endothall in some lakes in the USA, where adaption of the microbial communities in the lakes to use endothall as a carbon source has been hypothesized as a potential explanation (Michael Netherland, US Army Core of Engineers, pers. comm.). Where it is important to minimize off-site movement of endothall-treated water, faster degradation rates are desirable. If the microbes responsible for degradation of endothall can be isolated and cultured, then we may be able to pre-emptively inoculate areas with pre-adapted microbes to rapidly degrade endothall from these areas.

Temperature (together with moisture content) is the most important environmental factor affecting microbial growth and activity in soils [35]. As degradation of endothall is a biologically driven process, we expect that temperature will therefore also have a large influence on degradation rates. We are not aware of any other studies relating temperature and endothall degradation in the aquatic environment. However, several studies investigating the degradation of other microbially degraded herbicides when applied to soil report a positive relationship with temperature [36,37,38]. Characterization of this for endothall will be the subject of further investigations.

From a management perspective, the precise degradation rate of endothall in a waterbody will be difficult to predict because degradation rate varies according to sediment source, pre-exposure to endothall, endothall formulation, and likely temperature. Despite this, the general trend of degradation and persistence were similar and predictable.

In our study, when sediment is present most isomer-1 endothall persisted in the water column for 7–14 days. While this persistence time is comparable with some previous studies [29,30], reported persistence times vary widely (see reviews [16,22,23,24]). Persistence of isomer-2 was much longer but there are no prior studies to compare it to. Field studies are necessary to confirm if results of this mesocosm study are applicable in the natural waterbodies that are normally deeper and more dynamic than laboratory mesocosms.

This study provides useful insight into safer and better aquatic plant management using endothall. The findings of this study can be used by waterbody managers and regulatory authorities to better understand endothall decay in a range of aquatic environments. This understanding will underpin strategies to manage endothall-treated water, so that it does not move into sensitive, downstream environments. Further, this understanding can be used by these organizations to initiate studies to resolve the critical knowledge gaps we have identified, e.g., the relative herbicidal properties of the isomers, the time required for complete degradation of endothall and the mechanisms involved in endothall decay.

## 5. Conclusions

In conclusion, we provide direct evidence that the presence and characteristics of sediment are of key importance in the degradation of endothall in an aquatic environment, and we provide prima facie evidence that this importance is associated with the microbial community in the sediment. We have also identified that monoamine endothall has two sperate isomers that have different degradation characteristics. The herbicidal properties of these need to be investigated.

## Figures and Tables

**Table 1:** Description of treatments (source of water; presence/absence and source of sediment; endothall formulation; microbe augmentation). Endothall was applied at 2.4 mg ae L^−1^; Monoamine endothall was added as Teton™, Dipotassium endothall was added as Cascade™. The sources of water and sediment, and microbe augmentation are described in the text.

Treatment No.	Water	Sediment	Endothall Form	Microbe Augmentation
1	Potable	-	-	-
2	Coleambally	-	-	-
3	Central Goulburn	-	-	-
4	Potable	Garden	-	-
5	Coleambally	Coleambally	-	-
6	Central Goulburn	Central Goulburn	-	-
7	Potable	-	Monoamine	-
8	Coleambally	-	Monoamine	-
9	Central Goulburn	-	Monoamine	-
10	Potable	Garden	Monoamine	-
11	Coleambally	Coleambally	Monoamine	-
12	Central Goulburn	Central Goulburn	Monoamine	-
13	Potable	-	Dipotassium	-
14	Coleambally	-	Dipotassium	-
15	Central Goulburn	-	Dipotassium	-
16	Potable	Garden	Dipotassium	-
17	Coleambally	Coleambally	Dipotassium	-
18	Central Goulburn	Central Goulburn	Dipotassium	-
19	Potable	Garden	-	+
20	Coleambally	Coleambally	-	+
21	Central Goulburn	Central Goulburn	-	+
22	Potable	Garden	Monoamine	+
23	Coleambally	Coleambally	Monoamine	+
24	Central Goulburn	Central Goulburn	Monoamine	+

**Table 2:** Water quality data taken over the duration of the experiment. Values are mean ± one standard deviation. EC = electrical conductivity; DO = dissolved oxygen. All treatments with the same combination of water and sediment are grouped.

Treatment Grouping	Temperature (°C)	Turbidity (NTU)	EC (µS/cm^2^)	pH	DO (mg/L)
Potable water	17.5 ± 1.7 *	2 ± 1	97 ± 14	8.0 ± 0.2	9.5 ± 0.3
Coleambally water		22 ± 3	175 ± 16	7.9 ± 0.2	9.4 ± 0.3
Goulburn Valley water		22 ± 4	85 ± 10	8.0 ± 0.1	9.5 ± 0.3
Potable water + sediment		5 ± 4	423 ± 85	8.1 ± 0.1	9.0 ± 0.4
Coleambally water + sediment		48 ± 5	161 ± 24	8.2 ± 0.2	8.8 ± 0.3
Goulburn Valley water + sediment		35 ± 8	97 ± 23	8.1 ± 0.2	8.9 ± 0.3

* = Since all the mesocosm tubs were placed in one temperature-controlled glasshouse, temperature was not recorded separately for each treatment.

**Table 3:** Properties of the sediment used in the experiment. CEC = Cation exchange capacity.

Property	Central Goulburn Irrigation Channel	Coleambally Irrigation Channel	Garden Soil
pH (1:5 water)	5.4	6.2	7.3
Total soluble salt (ppm)	52.47	77.88	574.2
Total organic matter (%)	2.23	2.48	17.9
CEC (meq/100 of soil)	12.98	27.4	33.3
Colour	Yellowish brown	Light brownish grey	Dark grey
Texture	Light clay	Medium clay	Sandy clay loam light
Total active biological population (cfu/g soil) *	105,200	62,200	864,200

* = sum of active acetic acid bacteria, active fungi, cellulose utilizers, active yeasts, active actinomycetes, active photosynthetic bacteria.

**Table 4:** Analysis of variance for treatment effects on endothall isomer-1 concentration at day-0. DF = Degrees of freedom.

Effects	DF	F Value	*p* Value
Endothall form (adjusted for sediment)	1, 30	572.35	4.3 × 10^−21^
±sediment (adjusted for endothall form)	1, 30	16.64	0.00031
Interaction of endothall form and treatment	1, 30	12.12	0.0016
Further effects of treatment	11, 30	0.72	0.71

**Table 5:** Analysis of variance for treatment effects on endothall isomer-1 as % decline between day-0 and day-21. The data is analyzed after a log10(1.1-(%decline/100)) transformation. Treatment 14 is excluded from analysis (see methods). DF = Degrees of freedom.

Effects	DF	F Value	*p* Value
± sediment	1, 28	114,447	4.1 × 10^−52^
Within no sediment:			
Endothall form (monoamine vs. dipotassium)	1, 28	12.92	0.0012
Water source within endothall	3, 28	2.86	0.055
Within sediment added:			
Type of sediment (channel vs. garden)	1, 28	241.11	2.7 × 10^−15^
Endothall form (monoamine non-augmented vs. monoamine augmented vs. dipotassium)	2, 28	264.34	8.6 × 10^−16^
Interaction of type of sediment and endothall form	2, 28	2.78	0.079
Channel sediment source (Coleambally vs. Goulburn Valley)	1, 28	1.25	0.27
Interaction of endothall form and channel sediment source	2, 28	0.29	0.75

**Table 6:** Effect of endothall formulation and microbe augmentation, with and without sediment, and of sediment source on endothall isomer-1 concentration at day-21, as % decline from isomer-1 at day-0. A negative % decline is a percentage increase. Treatment 14 is excluded from analysis (% decline concentrations for 3 replicates of treatment 14: −13.0, 17.1 and 31.5). SED = Standard error of the difference.

Treatment	Log_10_(1.1-(%Decline/100)) (Transformed)	% Decline (Back Transformed)
Within no sediment:		
Monoamine endothall	0.109	−18.6
Dipotassium endothall	0.127	−23.9
Within sediment added:		
Monoamine non-augmented	−0.841	95.6
Monoamine augmented	−0.861	96.2
Dipotassium (non-augmented)	−0.935	98.4
SED	0.0043−0.0048	
Sediment source:		
Irrigation channel	−0.899	97.4
Garden	−0.841	95.6
SED	0.0037	

**Table 7:** Analysis of variance for isomer-2 at day-21, as % decline at day-0. The data is analyzed after a rank with ties transformation on treatments with monoamine endothall. All *p* values are calculated using permutation tests on the F statistic on rank transformed data. DF = Degrees of freedom.

Effects	DF	F Value	*p* Value
± sediment	1, 18	132.99	<0.00001
Within no sediment:			
Water source within endothall form	2, 18	0.28	0.76
Within sediment added:			
Type of sediment (channel vs. garden)	1, 18	0.27	0.61
Endothall form (monoamine non-augmented vs. monoamine augmented vs. dipotassium)	1, 18	44.33	0.00004
Interaction of type of sediment and endothall form	1, 18	0.76	0.40
Channel sediment source (Coleambally vs. Goulburn Valley)	1, 18	0.82	0.37
Interaction of endothall form and channel sediment source	1, 18	0.25	0.62

**Table 8:** Effect of treatment on the percent decline of isomer-2, from day-0 to day-21. SED = Standard error of the difference.

Treatment	Rank Transformed	Back Transformed
Within no sediment:		
Monoamine endothall	5.0	−22
Within sediment added:		
Monoamine non-augmented	14.0	54
Monoamine augmented	23.0	100.0
SED	1.35	

**Table 9:** Effect of treatments on the first day in which endothall isomer-1 concentration was observed to decay by 25, 50 or 75% of its total decay. Analysis only includes mesocosms treated with sediment. Days are transformed to ranks with ties. BT = Back transformed. SED = Standard error of the difference.

	25% Reduction		50% Reduction		75% Reduction	
	Rank	BT (day)	Rank	BT (day)	Rank	BT (day)
Coleambally:						
Dipotassium non-augmented	19.7	7	18.5	9	15.5	9
Monoamine non-augmented	24.0	9	18.5	9	18.0	9
Monoamine augmented	8.0	5	9.0	7	12.8	9
Central Goulburn:						
Dipotassium non-augmented	13.3	5	23.3	9	25.0	11
Monoamine non-augmented	8.0	5	21.2	9	21.5	9–11 *
Monoamine augmented	5.7	5	5.0	5	7.2	7
Garden:						
Dipotassium non-augmented	21.8	9	18.5	9	15.5	9
Monoamine non-augmented	17.5	7	9.0	7	7.5	7
Monoamine augmented	8.0	5	3.0	5	3.0	5
SED	3.10		1.95		3.04	

* = Back-transformation function is discontinuous at this point.

**Table 10:** Analyses of variance for treatment effects on the first day in which endothall isomer-1 had decayed by 25, 50 or 75% of its total decay. Analysis only includes mesocosms with sediment. Days are transformed to ranks with ties. All *p* values are calculated using permutation tests on the F statistic on rank transformed data. DF = Degrees of freedom.

Effects	DF	25% Reduction		50% Reduction		75% Reduction	
		F Value	*p* Value	F Value	*p* Value	F Value	*p* Value
Source of sediment	2	12.02	0.00090	17.90	0.00015	14.86	0.00026
Augmented vs. non-augmented	1	42.99	0.00002	164.10	<0.00001	39.17	<0.00001
Interaction of source of sediment and augmentation	2	2.94	0.08	6.06	0.011	5.46	0.016
Endothall form within non-augmented	1	0.99	0.33	11.91	0.0033	2.93	0.10
Interaction of source of sediment and endothall form within non-augmented	2	2.94	0.080	6.51	0.0079	3.01	0.076

## Supplementary Materials

Supplementary File 1
